# Inhalation in Pulmonary Tuberculosis

**Published:** 1886-07-20

**Authors:** 


**Inhalation in Pulmonary Tuberculosis.**—Renzi presents the following results of numerous observations upon the treament of pulmonary tuberculosis by medicated vapors:

1. Inhalations of minute quantities of the vapor of iodine, and of a mixture of iodoform and turpentine, induce amelioration of the local condition and of nutrition, but do not modify the fever, diarrhœa, or night-sweats. The mixture last named consists of twenty-five parts of turpentine to one of iodoform. Two to six drops of this are to be hourly volatilized in an apartment of average size.

2. Inhalations of sulphuretted hydrogen (four cubic inches of the gas to each cubic yard in the room) and of sulphurous acid augment the general and dynamo-metric strength of the patient, and have a beneficial effect upon nutrition and renal excretion. The sulphuretted hydrogen possesses an especial action upon the respiration, which, together with cough, is diminished in frequency.—*Review de Therapeutique*, March 1, 1886.

